# Leveraging human expert image annotations to improve pneumonia differentiation through human knowledge distillation

**DOI:** 10.1038/s41598-023-36148-7

**Published:** 2023-06-06

**Authors:** Daniel Schaudt, Reinhold von Schwerin, Alexander Hafner, Pascal Riedel, Christian Späte, Manfred Reichert, Andreas Hinteregger, Meinrad Beer, Christopher Kloth

**Affiliations:** 1 Department of Computer Science, Ulm University of Applied Science, Albert-Einstein-Allee 55, 89081 Ulm, Baden-Wurttemberg Germany; 2 Institute of Databases and Information Systems, Ulm University, James-Franck-Ring, 89081 Ulm, Baden-Wurttemberg Germany; 3 Department of Radiology, University Hospital of Ulm, Albert-Einstein-Allee 23, 89081 Ulm, Baden-Wurttemberg Germany

**Keywords:** Computer science, Information technology, Respiratory tract diseases, Machine learning, Predictive medicine

## Abstract

In medical imaging, deep learning models can be a critical tool to shorten time-to-diagnosis and support specialized medical staff in clinical decision making. The successful training of deep learning models usually requires large amounts of quality data, which are often not available in many medical imaging tasks. In this work we train a deep learning model on university hospital chest X-ray data, containing 1082 images. The data was reviewed, differentiated into 4 causes for pneumonia, and annotated by an expert radiologist. To successfully train a model on this small amount of complex image data, we propose a special knowledge distillation process, which we call Human Knowledge Distillation. This process enables deep learning models to utilize annotated regions in the images during the training process. This form of guidance by a human expert improves model convergence and performance. We evaluate the proposed process on our study data for multiple types of models, all of which show improved results. The best model of this study, called PneuKnowNet, shows an improvement of + 2.3% points in overall accuracy compared to a baseline model and also leads to more meaningful decision regions. Utilizing this implicit data quality-quantity trade-off can be a promising approach for many scarce data domains beyond medical imaging.

## Introduction

Having fast and reliable ways to screen infected patients is a learning from the COVID-19 pandemic. Developing machine learning models to assist clinical decision making in the beginning of a pandemic can be critical as it can shorten time-to-diagnosis and support specialized medical staff in an emergency setting^1^. A major hindrance to quickly building models and reacting to new infectious diseases is the restricted availability of (quality) data. This applies to the medical domain in general, where gathering large amounts of data is often difficult due to privacy concerns or high costs. This facilitates the need to leverage scarce data in a reasonable way.

Despite having methods like transfer learning and self-/semi-supervised learning, the performance of deep learning models depends significantly on the quantity of available data, as shown theoretically^2,3^ and empirically^4–6^. In this study we present such a case with limited amounts of data in the medical domain. We analyze chest X-ray (CXR) images of 4 different causes for pneumonia, as well as healthy patients, with as little as 74 images for viral/non-COVID-19 cases. It is our aim to leverage human expert knowledge to get medically adequate predictive results, despite working with scarce data.

For this purpose, we analyze COVID-19, other viral, fungal and bacterial pneumonia images. This makes the data quite complex and non-trivial to differentiate. To still achieve medically adequate performances, we leverage high quality annotated data to improve our classification model in a special knowledge distillation process. We dubbed our novel approach *Human Knowledge Distillation*. This process allows human experts to provide guidance during model training to improve performance and convergence, which is especially helpful in domains with very limited amounts of data. We demonstrate the usefulness of this approach by comparing different model types and architectures trained with Human Knowledge Distillation, all of which show improved performances compared to their respective baselines. We further examine the classification performance of the best resulting model, which we call *PneuKnowNet*. Compared to the respective baseline model, PneuKnowNet is able to adequately differentiate between 4 pneumonia classes in the presented study data.

In addition to this relevant application of Human Knowledge Distillation, we see many possible applications in further image domains with limited amounts of data. In summary, our main contributions are:

- We propose a novel approach, Human Knowledge Distillation, as a combination of feature-based knowledge distillation and consistency regularization. This approach enables deep learning image models to implicitly learn from human annotations on images to improve performance.
- We demonstrate the beneficial effect of our approach on CXR images to train a model that is able to differentiate between 4 causes for pneumonia as well as healthy patients as an example. The resulting models show significant improvements compared to their baselines, especially regarding the detection of specific pneumonia classes.
- We validate our approach for multiple different model architectures and training configurations, most of which show improved results compared to their respective baselines. We also examine the effect of a reduction of annotation data to potentially reduce annotation efforts.

## Related work

### Pneumonia detection

There exist many works applying deep learning to CXR images to detect a COVID-19 pulmonary disease^7–12^ or pneumonia in general^13–15^. However, most of these works use large publicly available CXR and COVID-19 image datasets. Most of these images are collected from heterogeneous sources with varying image and label quality, which raises concerns about the quality and valid evaluation of deep learning models^16,17^. Furthermore, they work with much more image data, often exceeding the data for this study by a factor between 10 and 100, while simultaneously only looking at a very limited number of pneumonia classes (mostly just two). For this study we analyze high quality, homogeneous image data from a single source and we differentiate between 4 causes for pneumonia and healthy cases.

### Human knowledge distillation

Using human knowledge to guide deep learning models is especially common in interactive image segmentation^18–21^. These models use human interactions (clicks or scribbles) to guide segmentation models towards the correct segmentation of regions. While these methods show very promising results, they focus on segmentation tasks. We on the other hand, want to improve classification tasks.

Zhang et al.^22^ use human categories for wrongly classified dermoscopic images to evaluate possibilities to improve classification models with human expertise. Jadhav et al.^23^ use knowledge learned from X-ray reports to improve a deep learning model’s performance on chest X-ray images. While both works try to achieve a goal similar to our approach, they do not use annotations on the image to guide the deep learning model with localization information. Zagoruyko et al.^24^ uses attention maps in a knowledge distillation process to improve a student model, but without using human-made annotations. This is achieved by Fukui et al.^25^ and Mitsuhara et al.^26^, who employ attention branch networks to manually edit visual explanations to embed human knowledge into classification models. Compared to our work, these works focus on editing the resulting attribution map and not the image itself.

Our Human Knowledge Distillation process can be understood as a mixture of semi-/self-supervised learning consistency regularization^27–29^ and the teacher-student architecture commonly found in knowledge distillation^30^, specifically in feature-based knowledge distillation^24,31–37^. In knowledge distillation the goal is typically to extract a condensed version of a big and cumbersome teacher model to reduce computational load while preserving almost identical performances. In our approach, both teacher and student model can have the same architecture and be of small size as well. Our goal is to simply learn an implicit representation for explicitly modified data. We take inspiration from Sohn et al.^38^, where weakly and strongly augmented variants of the same image were used to train a model. Instead of using augmentations, our student model learns from an additionally annotated image variant. As opposed to semi-/self-supervised methods, this provides the model with higher quality information present on the image. Thereby, we aim for consistency between a raw image and its corresponding annotated region of interest (ROI) variant.

## Materials and methods

To demonstrate the effect of Human Knowledge Distillation, we train a deep learning model to differentiate between 4 causes for pneumonia as well as healthy patients based on chest X-ray images from local university hospital study data. This section explains the origin and distribution of the data, as well as the deep learning model and Human Knowledge Distillation process.

### Data

**Figure 1:**
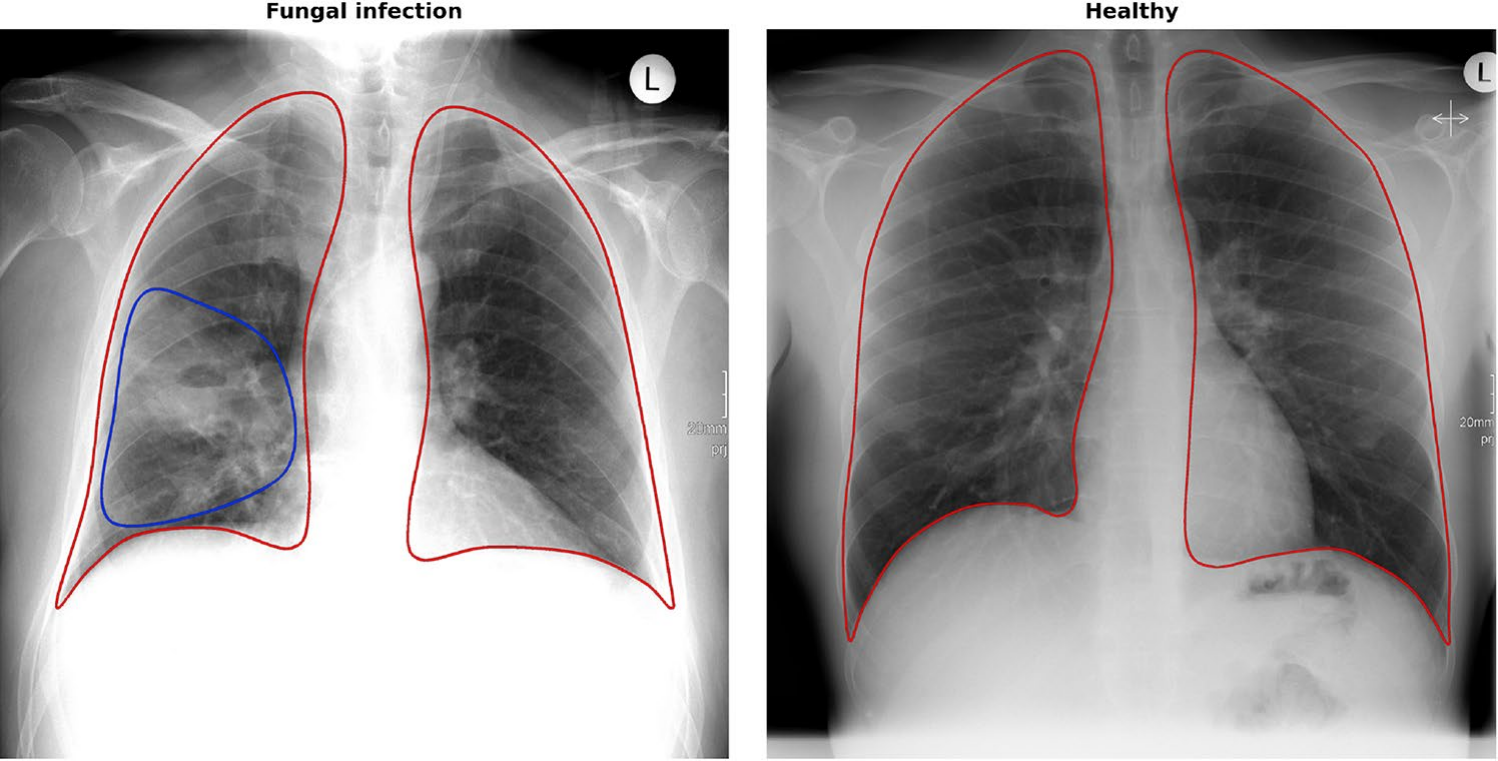
Chest X-ray ROI images with lungs marked in red. (Left) Fungal infection, typical ground-glass opacification marked in blue (ROI) All ROI annotations of this study show only the boundary of the infection, instead of a complete mask and use the same blue color regardless of pneumonia class. (Right) Healthy patient image.

The dataset specific to this single-center retrospective analysis consists of 1082 chest X-ray images from a total of 828 patients (342 female and 486 male) with ages ranging from 18 to 89 years (mean age 52.52 ± 17.45 years). These patients had chest radiography examinations due to their clinical symptoms. Radiographs were acquired on a portable flat detector (Flurospot Compact Siemens Healthcare, Erlangen Germany and DRX Evolution Carestream, Stuttgart Germany). The ethics board of the Medical Faculty and the University Hospital in Ulm approved this retrospective data evaluation study and waived the informed consent requirement (No. 271/20). All methods were carried out in accordance with relevant guidelines and regulations. Figure 1 shows two male patient example CXR images from our study data along with the relevant annotations.

#### Data acquisition

Radiographs were identified by retrospective database analysis of the local radiology department. Bacterial infections were proven using sample material collected by bronchoalveolar lavage or sputum. Fungal infections were confirmed by positive microscopy or cultured organisms. All patients with COVID-19 were confirmed by nasopharyngeal swabs followed by RT-PCR assay to confirm the diagnosis. Detection and verification of virus infect was done from bronchoalveolar lavage by real time PCR using a commercially available assay.

#### Data labeling

A dedicated thoracic radiologist (CK) with 9 years experience in lung imaging verified and relabeled the datasets. Images were differentiated and labeled as cases of 110 (10.17%) COVID-19 (C), 673 (62.20%) healthy patients (H), 100 (9.24%) bacterial infection (B), 125 (11.56%) fungal infection (F), and 74 (6.84%) other viral infection (V). Table 1 shows the demographic variables for training and validation cohorts used in this study.

**Table 1:** Summary of demographic variables and imaging protocol variables of CXR data for training and validation cohorts used in this study.

Variable	Group	Training data	Validation data
Age	mean ± std	51.75 ± 17.54	53.82 ± 18.05
	<20	20 (2.31%)	3 (1.38%)
	20–29	110 (12.72%)	29 (13.36%)
	30–39	105 (12.14%)	22 (10.14%)
	40–49	127 (14.68%)	28 (12.90%)
	50–59	191 (22.08%)	58 (26.73%)
	60–69	171 (19.77%)	26 (11.98%)
	70–79	102 (11.79%)	36 (16.59%)
	80–89	39 (4.51%)	15 (6.91%)
Sex	Male	533 (61.62%)	134 (61.75%)
	Female	332 (38.38%)	83 (38.25%)
Imaging view	AP	238 (27.51%)	68 (31.34%)
	PA	625 (72.25%)	149 (68.66%)

Age and sex statistics are expressed on a patient level, while imaging view statistics are expressed on an image level with anteriorposterior (AP) and posterioranterior (PA) views.

#### Data annotation

An Impax EE R 20 XVIII SU1 image archiving and communication system was applied for selecting the radiographs from the radiological database. A freehand drawing tool was used to segment the lung based on its anatomical landmarks. Furthermore, the pathological ROIs were marked, as shown in Fig. 1. These regions contain typical ground-glass opacifications, induced by pneumonia. The same blue color outline was used for all pneumonia classes. With the image archiving and communication system, the images were anonymized and exported as JPEG files and stored separately. Going forward, the original non-annotated images are called *raw images*, whereas the ROI annotated images are denoted as *ROI images*.

The quality of the presented dataset is unique with regard to its annotation detail. To the best of our knowledge, no openly available CXR dataset matches the freehand ROI annotations of this study data. Some openly available datasets do provide annotations in the form of bounding boxes^39^, which provide only coarse localization information.

#### Image preprocessing

The raw and ROI images have 3 RGB channels and a width between 2084 pixels and 4240 pixels with a mean of 2825.01 pixels, as well as a height between 1800 pixels and 4240 pixels with a mean of 3053.89 pixels. Raw images and their corresponding ROI version have the same size and only differ in their annotation. As input image size we keep the pretrained resolution of $224\times 224$ pixels. All images are resized with bilinear interpolation and normalized with the mean and standard deviation values from ImageNet^40^ images. Although the image space of this study is different from ImageNet, changing these values would interfere with the pretrained models. Raw and ROI images are treated equally with regards to preprocessing and augmentation steps. The ROI images are fed directly into the model in the same manner as the raw images, without using any segmentation mask, allowing for freehand expert annotations without using specific tools to extract masks. The input tensors are of shape [batchsize,channels,height,width], resulting in input dimensions of [8,3,224,224] in our experiments.

#### Evaluation splits

To evaluate our models, we use a holdout method. To avoid patient overlap between the splits, we use a random subject-based split based on patients with roughly 20% of images as validation data. We attempt to preserve the percentage of samples for each label as much as possible, given the constraint of non-overlapping patients between the splits. Table 2 shows the resulting label distribution for training and validation splits.

**Table 2:** Label distribution for training and validation splits.

Label	Training data (%)	Validation data (%)
Healthy	543 (62.77)	130 (59.91)
Fungal infection	96 (11.1)	29 (13.36)
COVID-19	87 (10.06)	23 (10.60)
Bacterial infection	81 (9.36)	19 (8.76)
Viral infection	58 (6.71)	16 (7.37)

### Human knowledge distillation

**Figure 2:**
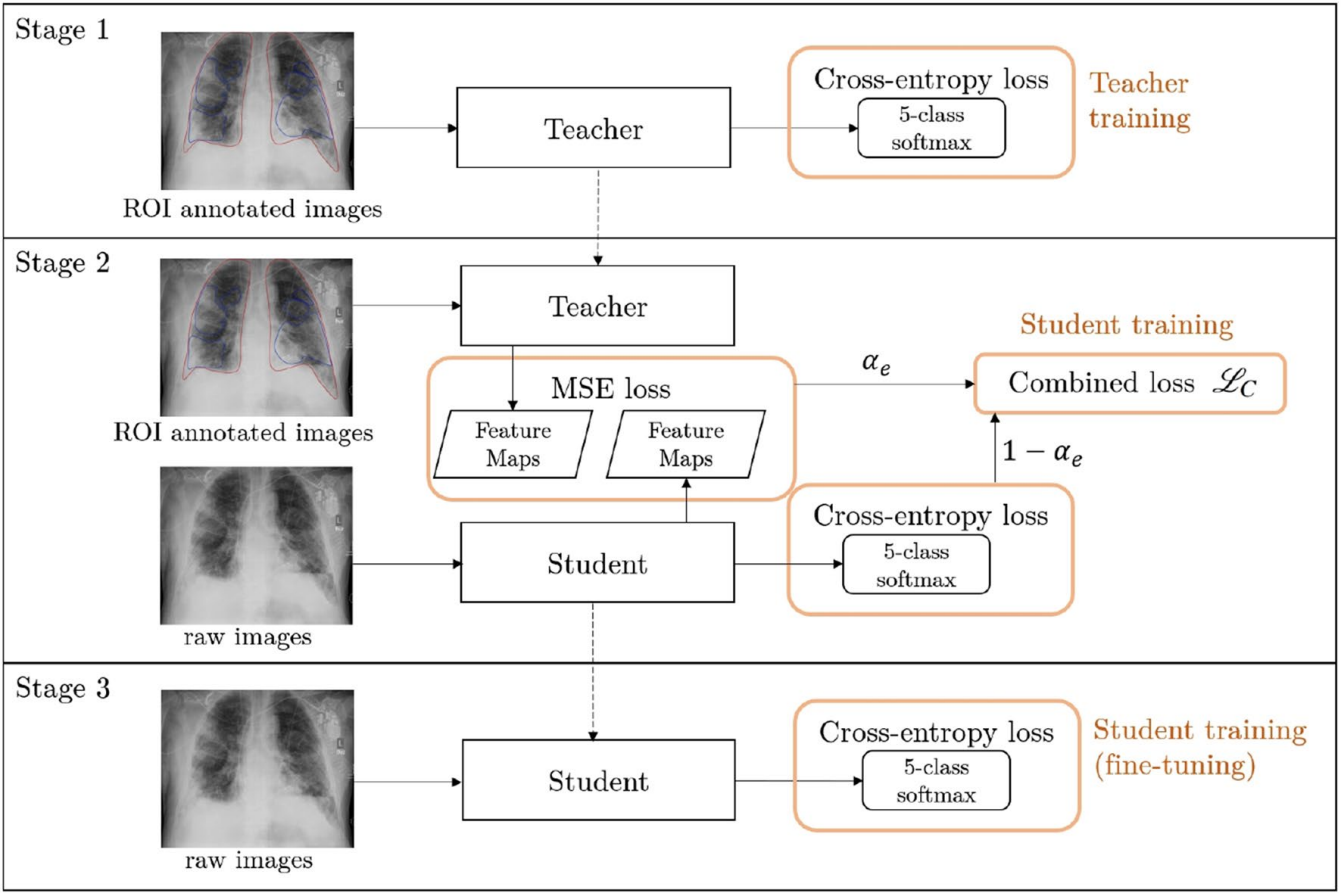
Overview of our Human Knowledge Distillation training process, demonstrated here with medical ROIs as annotated images and 5 classes for pneumonia differentiation. (Stage 1) Teacher training on ROI images. (Stage 2) Student training with consistency regularization from the teacher model by using ROI images. (Stage 3) Student training without consistency regularization on raw images.

We employ our Human Knowledge Distillation process in 3 stages: **teacher training**, **teacher-student training**, and **student fine-tuning**, as shown in Fig. 2. In the first stage, a teacher model is trained on annotated images that present complete localization information. In the second stage, a student model is trained on raw images with an additional consistency regularization from the teacher model of a corresponding annotated image. Thereby, the student model indirectly learns to use this localization information through the teacher model. In the last stage, the student model is fine-tuned in a standard classification pipeline without using consistency regularization. This process enables the final student model to implicitly utilize localization information in a human-guided fashion, thus indirectly applying it during inference on raw images. The application demonstrated in this work employs medical ROIs on CXR images as annotations to learn from. We call our final model PneuKnowNet.

#### Stage 1 (Teacher training)

In this stage we train a Convolutional Neural Network on the annotated ROI images. Thus, the model has access to localization information of pathogenic ROIs and the outline of the lung. This stage can be understood as a *human-guided training*, where we point the model towards areas of the image that a human expert deems important. Using this additional information, we expect the teacher model to perform well, even early in the training process. Note, that the ROIs only provide localization information and do not reveal the label of a pathogenic image, i.e. the cause of the pneumonia, since all pneumonia positive images use the same blue color outline. The weights of the teacher model are fixed after this stage and not trained any further during our process.

#### Stage 2 (Teacher-student training)

In this stage we *distill* the knowledge of the teacher model $f_t$ for its use in a student model $f_s$, which thereby learns to look for pertinent information in the important regions. To achieve this, we define a combined loss function $\mathcal {L}_C$ using a weighted sum of the consistency loss and the classification loss with weight $\alpha _e$. We adapt the weight $\alpha _e$ for each epoch $e \in \{0,\ldots ,E_\text {distill},\ldots ,E_\text {total}\}$ linearly during training between 0 and 0.5:

1 $$\begin{aligned} \mathcal {L}_C&= \alpha _e \cdot \text {MSE}\left( f^{(-1)}_{t}(x_\text {ROI}),f^{(-1)}_{s}(x_\text {raw})\right) +(1-\alpha _e) \cdot \text {CE}\left( f_s(x_\text {raw}),Y\right) , \text {with} \end{aligned}$$

2 $$\begin{aligned} \alpha _e&= {\left\{ \begin{array}{ll} \frac{1}{2} \cdot \left( 1 - \frac{e}{E_\text {distill}}\right) &{} \text {if } e<E_\text {distill},\\ 0 &{} \text {otherwise}. \end{array}\right. } \end{aligned}$$

    The consistency loss is calculated by using the mean squared error (MSE) between the feature maps of the last convolutional layer $f_m^{(-1)}$ for $m \in \{s,t\}$ of the teacher and student model. Integrating feature maps into a knowledge distillation process to improve student model learning is a known approach and has been explored in numerous ways^24,31–37^ with similar loss functions. In this work we use two different image variants to motivate a consistency loss component similar to semi-supervised-learning approaches^27–29^. While the student model receives raw images $x_\text {raw}$ as input, the teacher model uses the corresponding ROI images $x_\text {ROI}$. As for the classification loss, we use cross-entropy (CE) between softmax model output and ground truth labels *Y*. $E_\text {distill}$ is a hyperparameter, that specifies the amount of epochs in stage 2, and as such, the amount of epochs for the teacher-student training. We start with a balanced loss function and reduce the influence of the consistency component during training. This way, the student model receives strong guidance at the beginning of the training process, while also needing to adapt to the raw images towards the end of the training.

#### Stage 3 (Student fine-tuning)

In this stage the consistency regularization component vanishes due to *e* exceeding $E_\text {distill}$ and $\alpha _e$ subsequently becoming 0. Without any guidance from the teacher model, the student is being fine-tuned on raw images only. After this final training stage, the student model $f_s$ can now be used for inference.

#### Training details and configurations for pneumonia differentiation

We demonstrate the effect of Human Knowledge Distillation on our presented CXR study data for pneumonia differentiation. To validate our approach, we train multiple model architectures with this process: ResNet50^41^, EfficientNet-B0^42^, EfficientNet-B1^42^, ConvNeXt-T^43^, and ConvNeXt-S^43^. Since we want to focus on our Human Knowledge Distillation process, we are not overly concerned with the type or architecture of the selected models themselves. Therefore, we present a broader selection of older and newer state-of-the-art models, which have been used extensively in academic literature. All experiments were repeated 5 times to increase the robustness of our results.

We train baseline models for all architectures and configurations to compare our Human Knowledge Distillation models as a point of reference. These models use the same architectures and hyperparameter settings as our knowledge distillation models and are trained in a standard end-to-end pipeline on the raw images of our CXR study data.

All models have been pretrained on the ImageNet^40^ database. This allows us to use finely calibrated weights as a starting point for our training. Contrary to traditional transfer learning, we do not freeze any weights for the training process, but use all gradients for updates. This is to compensate for the shift in image distributions between the pretraining data and our CXR data. ImageNet depicts a diverse dataset with 1000 classes and has therefore a very different image space compared to the desaturated CXR images of this study. We replace the final layer with a linear layer of 5 output nodes, one for each class.

Furthermore, we use image augmentation pipelines to artificially increase the size of the training data and reduce overfitting during model training. To examine the effects of augmentations on our method, we consider 2 different pipelines. Table 3 shows a strong and a weak augmentation pipeline. The weak augmentation pipeline consists only of a resize operation and an affine transformation. This pipeline should preserve the nature of the image and produce only slight variations. The strong augmentation pipeline includes the same transformations as the weak pipeline, but also introduces variations in brightness and contrast, as well as sharpen and blur operations. This pipeline was inspired by the winning solution to the 2021 SIIM-FISABIO-RSNA Machine Learning COVID-19 Challenge^44^. All augmentations are done via the Albumentations library^45^.

**Table 3:** Strong and weak augmentation pipelines.

Augmentation	Parameters	Probability
Strong augmentations		
Resize	height=224, width=224	1.0
ShiftScaleRotate	scale_limit = 0.5, rotate_limit = 0, shift_limit = 0.1	1.0
One of:		0.9
[CLAHE,	clip_limit = 4.0, grid_size = (8, 8)	1.0
RandomBrightnessContrast,	brightness_limit = 0.2, contrast_limit = 0.2, brightness_by_max = True	1.0
RandomGamma]	gamma_limit = (80, 120)	1.0
One of:		0.9
[Sharpen,	alpha = (0.2, 0.5), lightness = (0.5,1.0)	1.0
Blur,	blur_limit = 7	1.0
MotionBlur]	blur_limit = 7	1.0
One of:		0.9
[RandomBrightnessContrast,	brightness_limit = 0.2, contrast_limit = 0.2, brightness_by_max = True	1.0
HueSaturationValue]	hue_shift_limit = 20, sat_shift_limit = 30, val_shift_limit = 20	1.0
Normalize	mean = (0.485, 0.456, 0.406), std = (0.229, 0.224, 0.225)	1.0
Weak augmentations		
Resize	height = 224, width = 224	1.0
ShiftScaleRotate	scale_limit = 0.5, rotate_limit = 0, shift_limit = 0.1	1.0
Normalize	mean = (0.485, 0.456, 0.406), std = (0.229, 0.224, 0.225)	1.0

Augmentations carried out with Albumentations library^45^.

**Figure 3:**
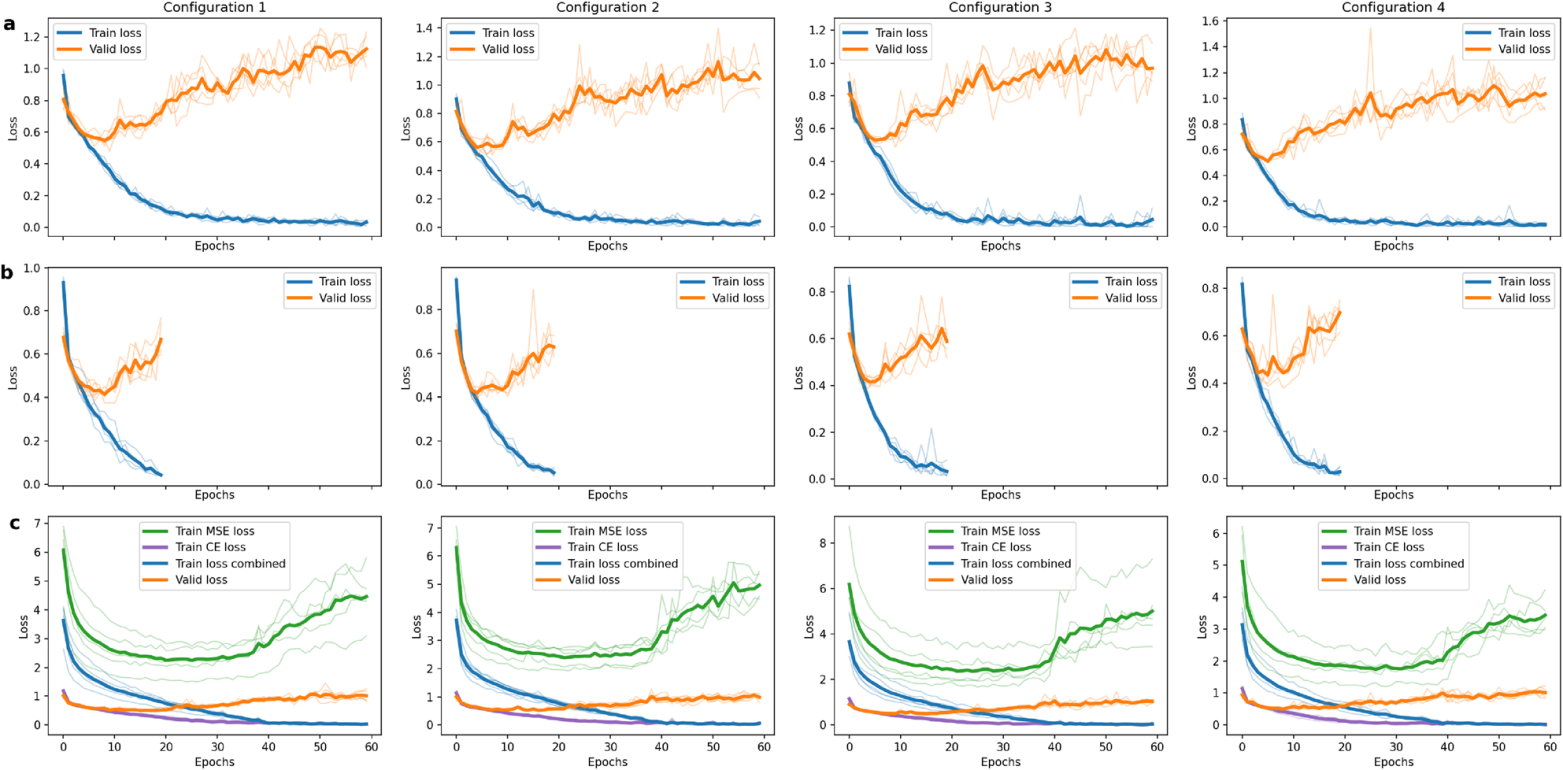
Training and validation loss curves for ConvNeXt-S baseline (**a**), teacher (**b**), and student (**c**) models. Bold lines represent the mean of 5 repeated runs.

We pair these augmentation pipelines with varying settings of dropout, since these hyperparameters can impact the performance of deep learning models significantly. We examine our method with 4 different configurations of dropout and augmentations, as shown in Table 4. If used, dropout is applied before the classification layer with a probability of 0.5. While we alternate dropout and augmentation pipelines for baseline and student models, we keep dropout active for all teacher models. This is to weaken overfitting as seen in Fig. 3, which seems to appear faster with ROI images. Examining different configurations for augmentation and dropout works as an ablation study to show the robustness of our method, independently of changes to those impactful hyperparameters.

**Table 4:** Different training configurations for dropout and augmentations for baseline, teacher and student models.

Model	Configuration 1	Configuration 2	Configuration 3	Configuration 4
Baseline				
Dropout	0.5	None	0.5	None
Augmentations	Strong	Strong	Weak	Weak
Teacher				
Dropout	0.5	0.5	0.5	0.5
Augmentations	Strong	Strong	Weak	Weak
Student				
Dropout	0.5	None	0.5	None
Augmentation	Strong	Strong	Weak	Weak

Same settings apply for all evaluated model architectures.

All other hyperparameter settings for the baseline model and Human Knowledge Distillation models are shown in Table 5. We keep most of these hyperparameters constant for all trained models to validate the effect of our Human Knowledge Distillation process. To make the comparison between baseline and Human Knowledge Distillation models fair, we use the same amount of total training epochs ($E_\text {total}=60$ each). The amount of epochs are chosen as a generous upper bound for model improvement. In our experiments, the models diverge much faster than that, as shown by the loss curves in Fig. 3. This is especially true for teacher models, which we only train for 20 epochs respectively. All final models are selected from the epoch with the lowest validation loss. We use PyTorch^46^ to carry out the computations.

**Table 5:** Hyperparameter settings for baseline model and Human Knowledge Distillation models.

Hyperparameter	Baseline	Teacher	Student
Optimizer	Adam^47^	Adam^47^	Adam^47^
Loss function	Cross-entropy	Cross-entropy	$\mathcal {L}_C$ 2
Batchsize	8	8	8
Base learning rate	1e−4	1e−4	1e−4
Learning rate scheduler	Cosine decay	Cosine decay	Cosine decay
Distillation epochs ($E_\text {distill}$)	N/A	N/A	40
Total epochs ($E_\text {total}$)	60	20	60
Optimizer momentum	$\beta _1, \beta _2 = 0.9, 0.999$	$\beta _1, \beta _2 = 0.9, 0.999$	$\beta _1, \beta _2 = 0.9, 0.999$

Same settings apply for all evaluated model architectures.

### Ethical approval

The ethics board of the Medical Faculty and the University Hospital in Ulm approved this retrospective data evaluation study and waived the informed consent requirement (No. 271/20).

## Results

In this section we compare the results of our Human Knowledge Distillation training process with a baseline model for multiple model architectures on our CXR pneumonia differentiation study data. For the best performing model, we compare precision, recall, and F1-score for all 5 classes. We also examine the effect of reducing the amount of ROI images for the teacher model, which could potentially reduce annotation costs. Lastly, we compare the GradCAM activations^48^ of the models by leveraging the given ROIs to see which model is more in line with human expert decision regions. All metrics are being calculated on the validation data and reported as mean ±std of 5 independent runs.

Table 6 shows the overall accuracy for different model architectures and training configurations for all stages of our Human Knowledge Distillation process and their respective baseline models. Remarkably, 17 out of 20 different combinations of models and configurations show improvements using Human Knowledge Distillation. Only 3 combinations show reduced performances compared to their respective baseline. The remaining improvement ranges from + 0.19% points to + 3.23% points. Configurations 1 and 3 yield favorable results due to the application of dropout to reduce overfitting. Looking at the different configurations for augmentation and dropout as an ablation study, our method shows consistent improvements for different settings of these impactful hyperparameters.

The stage 1 teacher models consistently have the highest performance. This makes sense, since these models have access to the most information during training, provided by the ROIs. Still, Human Knowledge Distillation models seem to achieve almost the same performance, despite having no explicit access to the additional image information. It is important to note, that the teacher model can not be used for inference on raw, non-annotated images since the model learned to rely on the annotations to make predictions. Thus, we have successfully transferred knowledge to a model to be used in an implicit way when classifying new images without annotations.

**Table 6:** Overall accuracy (in %) for Human Knowledge Distillation process for different model architectures and configurations.

Model/Architecture	ResNet50	EfficientNet-B0	EfficientNet-B1	ConvNeXt-T	ConvNeXt-S
Configuration 1					
Baseline	78.25 ± 1.14	78.71 ± 1.22	77.60 ± 1.19	78.25 ± 1.47	78.53 ± 0.37
Teacher (Stage 1)	75.21 ± 1.63	83.32 ± 1.22	82.03 ± 1.96	81.57 ± 1.20	83.69 ± 1.64
Student (Stage 2 + 3)	**79.08** ± **0.99**	76.22 ± 1.99	**78.34** ± **1.24**	79.35 ± 1.47	**80.83** ± **1.03**
Improvement (in pp.)	+ 0.83	− 2.49	+ 0.74	+ 1.09	+ 2.3
Configuration 2					
Baseline	77.33 ± 1.47	76.22 ± 2.80	76.68 ± 1.86	76.22 ± 1.59	79.72 ± 1.57
Teacher (Stage 1)	72.90 ± 1.14	81.47 ± 0.18	83.50 ± 1.96	83.50 ± 1.44	82.12 ± 1.71
Student (Stage 2 + 3)	77.51 ± 2.09	79.17 ± 1.53	78.06 ± 1.66	79.45 ± 0.95	80.65 ± 1.54
Improvement (in pp.)	+ 0.18	+ 2.95	+ 1.38	+ 3.23	+ 0.93
Configuration 3					
Baseline	77.97 ± 0.84	77.97 ± 1.07	76.31 ± 2.11	78.16 ± 0.69	79.63 ± 1.14
Teacher (Stage 1)	76.41 ± 3.37	82.21 ± 1.15	80.83 ± 1.59	82.58 ± 2.45	82.49 ± 0.65
Student (Stage 2 + 3)	78.62 ± 1.15	**79.26** ± **1.72**	77.05 ± 0.98	**80.18** ± **1.05**	79.35 ± 1.44
Improvement (in pp.)	+ 0.65	+ 1.29	+ 0.74	+ 2.02	− 0.28
Configuration 4					
Baseline	79.45 ± 1.42	77.60 ± 1.29	75.39 ± 1.88	77.42 ± 2.04	79.35 ± 2.32
Teacher (Stage 1)	77.88 ± 2.96	82.58 ± 1.35	81.01 ± 1.28	82.40 ± 1.25	81.57 ± 1.51
Student (Stage 2 + 3)	78.89 ± 0.61	77.79 ± 2.66	76.13 ± 2.27	79.63 ± 0.94	80.74 ± 2.27
Improvement (in pp.)	− 0.56	+ 0.19	+ 0.77	+ 2.21	+ 1.39

Improvement of student models compared to baseline models in percentage points. All results are reported as mean ± std of 5 independent training runs. Significant values are in bold.

The best absolute performance is achieved by the ConvNeXt-S models in configuration 1 with 80.83% overall accuracy with Human Knowledge Distillation. We further examine more detailed classification metrics for these models. Table 7 shows precision, recall, and F1-score for baseline and student models. While the baseline model shows better precision for COVID-19, all other metrics favor the student model. While most improvements are minor, the increase in precision for the viral class is notable. The student models also show better recall values for all classes, which is especially important in this sensitive medical setting, since false-negatives would lead to undetected cases.

**Table 7:** Evaluation metrics for ConvNeXt-S baseline and student models on validation data.

Label	Precision		Recall		F1-Score	
	Baseline	Student	Baseline	Student	Baseline	Student
Bacterial	.3260 ± .0498	**.3650** ± **.0328**	.3474 ± .0976	**.3895** ± **.0714**	.3307 ± .0579	**.3734** ± **.0376**
COVID-19	.**8292** ± **.0573**	.7906 ± .0692	.7478 ± .0928	**.7826** ± **.0615**	.7793 ± .0296	**.7864** ± **.0644**
Healthy	.9641 ± .0028	**.9745** ± **.0074**	.9923 ± .0049	**.9954** ± **.0038**	.9780 ± .0016	**.9848** ± **.0033**
Fungal	.4946 ± .0141	**.5282** ± **.0185**	.5517 ± .0899	**.6483** ± **.0593**	.5179 ± .0386	**.5810** ± **.0294**
Viral (other)	.1329 ± .1097	**.2167** ± **.1944**	.1000 ± .0848	**.1125** ± **.1212**	.1136 ± .0949	**.1449** ± **.1487**

All results are reported as mean ± std of 5 independent training runs. Significant values are in bold.

Figure 3 shows the training and validation loss curves for ConvNeXt-S models. The baseline and teacher models show significant overfitting due to the low amount of data. The loss curves for the student model show a reduced overfitting effect. This could indicate an implicit regularization effect through the consistency loss. The MSE loss shows a significant increase after epoch 40, which is expected, as $E_\text {distill}=40$ was chosen. The different training configurations do not seem to influence the loss curves significantly.

**Figure 4:**
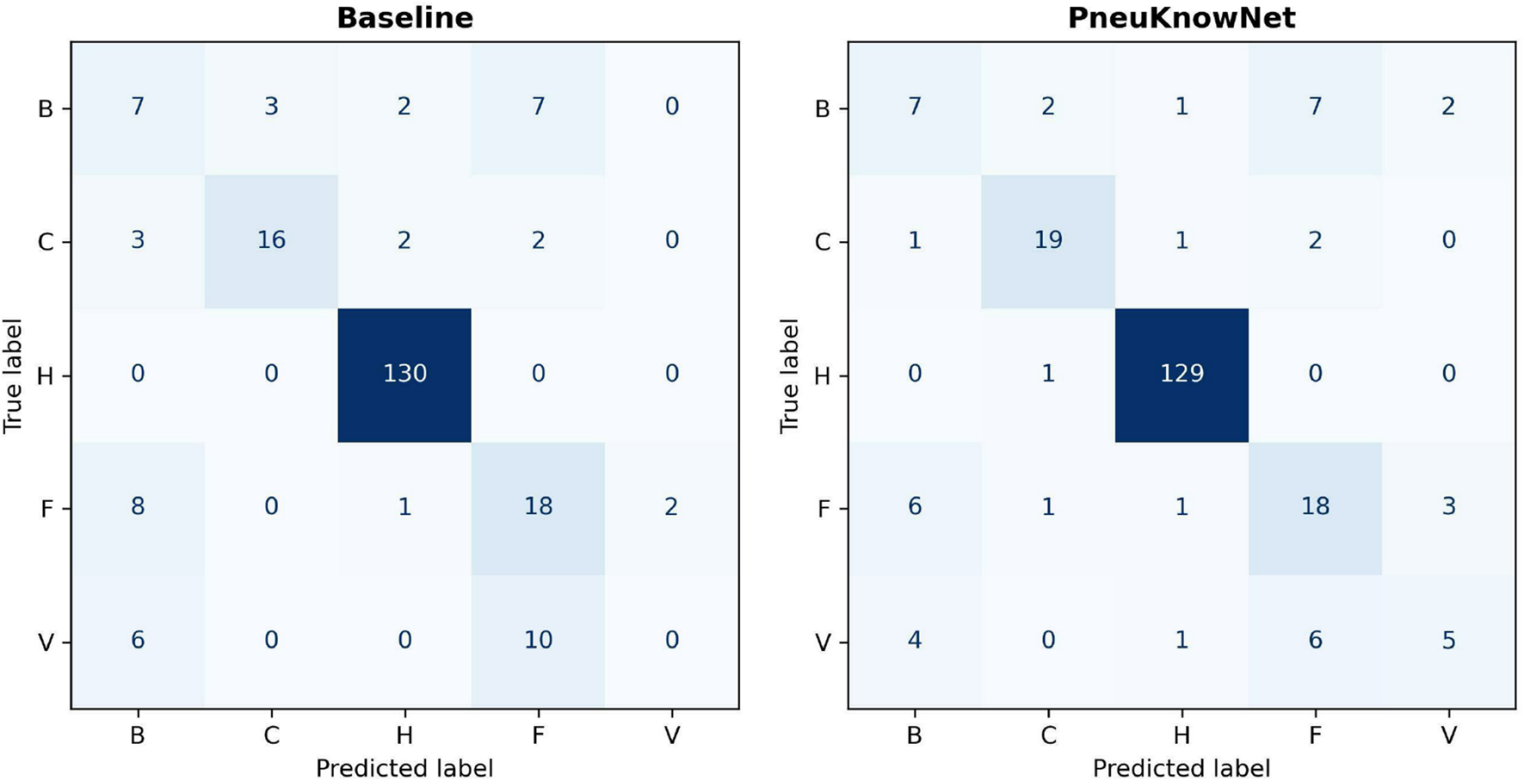
Confusion matrix for baseline model (left) and PneuKnowNet (right) on validation data.

We further examine the ConvNeXt-S baseline and student models with the lowest validation error out of the 5 repeated runs. We name this most promising student model PneuKnowNet. Figure 4 shows the confusion matrix for the baseline model and PneuKnowNet in absolute values for all 5 classes on the validation data. It is notable, that the baseline model does not predict any viral cases correctly, while PneuKnowNet does. Furthermore, the bacterial and fungal cases seem to get confused by both models, which are non-trivial to separate, even for human experts. In case of a binary decision (pneumonia vs healthy) PneuKnowNet achieves 97.70% accuracy.

### Reduction of annotation effort

In this study we use a dataset that is labeled with extra annotations by a human expert. For those cases where this labeling process is non-trivial and potentially costly, the amount of extra annotations might be limited. We therefore investigate the impact of a reduced amount of ROI images on our method. Table 8 shows the results for our ConvNeXt-S models when using only 10%, 30% or 50% of the available ROI images. These experiments use dropout and the strong augmentation pipeline (Configuration 1).

**Table 8:** Impact on performance with reduced amount of ROI images for ConvNeXt-S models.

ROI %	Model	Accuracy
10% ROI	Teacher	80.00 ± 2.57
	Student	78.25 ± 1.50
30% ROI	Teacher	82.03 ± 0.82
	Student	80.09 ± 1.11
50% ROI	Teacher	83.78 ± 0.54
	Student	80.28 ± 1.58
100% ROI	Baseline	78.53 ± 0.37
(for reference)	PneuKnowNet	80.83 ± 1.03

Interestingly, a model trained with only 10% of ROI images can almost achieve the same performance as our baseline model. The 30% ROI model surpasses the baseline by a significant margin and the 50% baseline model is only 0.55 percentage points behind the 100% ROI model (PneuKnowNet). This suggests, that positive training effects can still be achieved when using a fraction of the available data for extra annotations.

### Training teacher on raw images

We want to investigate whether the improvement of our Human Knowledge Distillation method stems from a transfer of knowledge of the infiltration areas, or is due to a regularizing effect of the distillation process. To verify the effectiveness of the presence of ROIs on images for our method, we train the teacher models on raw images instead. In this setup, no information about the presence and location of infiltration areas is introduced to the models, only the regularization effect of the distillation process remains. Table 9 shows the results for all evaluated models. These experiments use dropout and the strong augmentation pipeline (Configuration 1).

**Table 9:** Overall accuracy (in %) for Human Knowledge Distillation process on ROI images and raw images for different model architectures.

Model/Architecture	ResNet50	EfficientNet-B0	EfficientNet-B1	ConvNeXt-T	ConvNeXt-S
Baseline	78.25 ± 1.14	**78.71** ± **1.22**	77.60 ± 1.19	78.25 ± 1.47	78.53 ± 0.37
Student (ROI images)	**79.08** ± **0.99**	76.22 ± 1.99	**78.34** ± **1.24**	**79.35** ± **1.47**	**80.83** ± **1.03**
Student (Raw images)	77.51 ± 0.68	78.62 ± 1.71	77.88 ± 2.46	78.25 ± 1.76	78.80 ± 1.40
Change (in pp.)	− 1.57	+ 2.40	− 0.46	− 1.10	− 2.03

Change in accuracy between both student models in percentage points. Baseline model accuracy for reference. All results are reported as mean ± std of 5 independent training runs. Significant values are in bold.

Using raw images instead of ROI images to train the teacher models yields worse results for all model architectures except the EfficientNet-B0. For this specific architecture, using neither ROI images nor raw images shows any improvement over the baseline model. In all other cases, training the teacher models with ROI images leads to the described improvement of our Human Knowledge Distillation process compared to the baseline models. This is somewhat expected, since the teacher model can not learn and distill the additional information that comes from using the ROI images to the student model.

### Explainability

**Figure 5:**
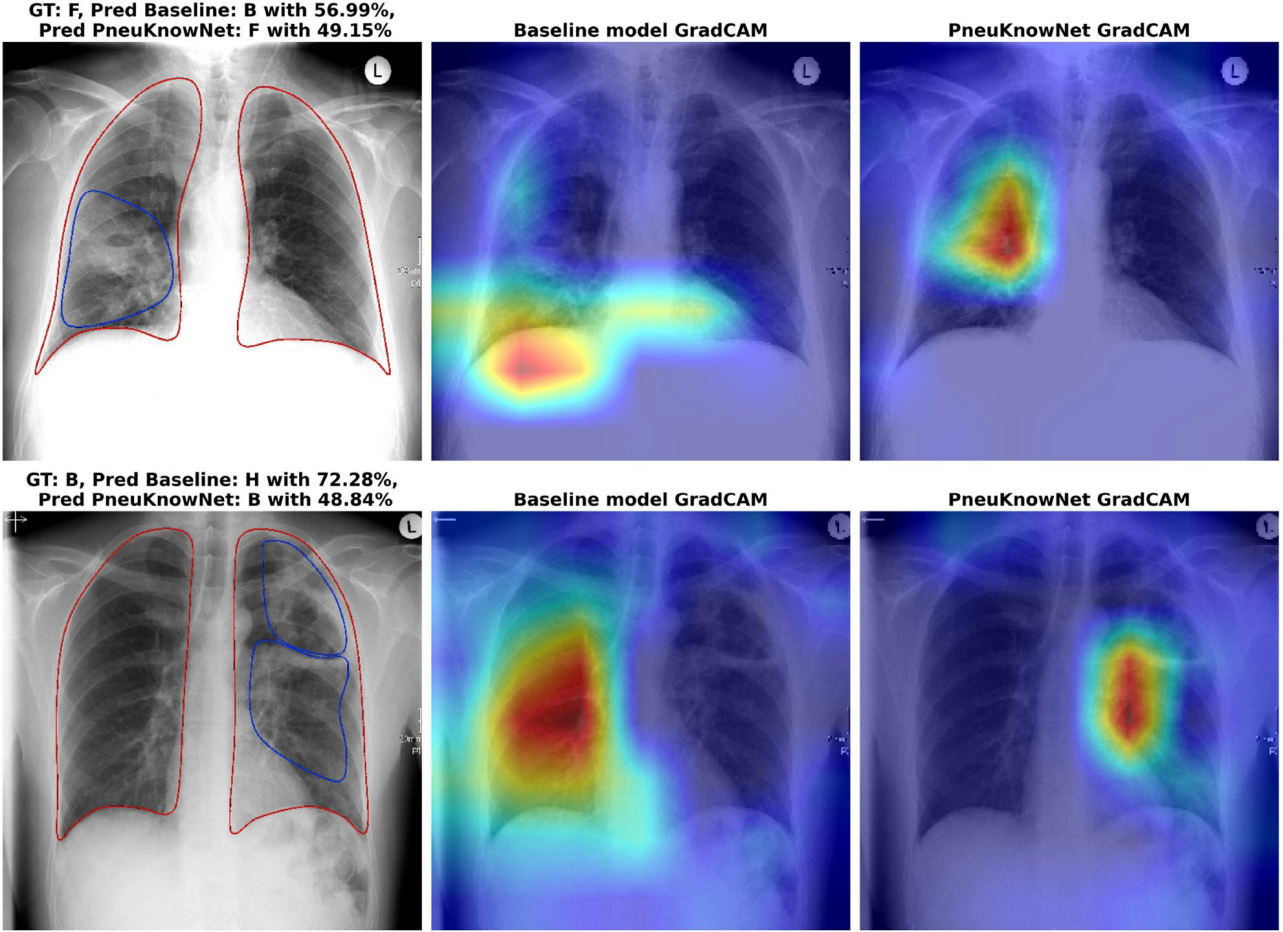
(Top row) GradCAM attribution for a bacterial pneumonia case, 56 years old male patient with fungal infection in the right lower lung. For this case, the attribution of the baseline model is more diffuse and not as clean as PneuKnowNet. Furthermore, the baseline model does not predict the fungal infection, but rather a bacterial infection. (Bottom row) GradCAM attribution for a bacterial case, 44 years old male patient with bacterial infection of the left apical lung. Peribronchial consolidation area with positive bronchopneumogramm and associated a little bit fluid in the pleura. In this instance, the baseline model incorrectly attributes the right lung. Both models make incorrect predictions, but the baseline model predicts a healthy patient, which would be detrimental.

While it is not the focus of this paper, we want to point out that our approach also lends itself well to the important aspect of explainability. The latter is of special interest in the health care domain. Using an attribution method like GradCAM, we can highlight important decision regions in the image^48^. Figure 5 shows two example classifications and corresponding GradCAM activations. Both images show a clear advantage for PneuKnowNet, which correctly identifies the relevant areas in both cases.

## Limitations and discussion

In this work, we presented a novel training process, Human Knowledge Distillation, which enables deep learning image models to implicitly learn from additional human-made annotations on the images. This can be especially useful for domains with very limited amounts of data available and presents an opportunity for a data quality-quantity trade-off to improve model performance and enable better convergence. We demonstrate the positive effects on performance on our CXR study data to differentiate between 4 causes of pneumonia, as well as healthy patients as an example. We showed that our Human Knowledge Distillation models do not only perform better than a baseline classification pipeline in regards to classification metrics, but also seem to be more consistent with human decision regions. We evaluated our results on multiple model types and architectures, as well as training configurations, all of which show improved performances with our training approach. We also examined a reduction in the amount of ROI images to potentially reduce annotation costs. Therefore, we presented a method to obtain a potent and trustworthy model for scarce data domains.

Our method requires the training of multiple deep learning models in a more complex pipeline, making the approach computationally more expensive. Since this method is specifically tailored towards scenarios with small sample sizes and therefore short training cycles, this seems an acceptable trade-off. Depending on the level of detail, creating the extra annotations can be quite costly and/or time-consuming, although we showed, that a reduced amount of annotations could still be serviceable. In our demonstration, medical ROIs were used as annotations, but further annotation techniques could be explored. The introduced loss function $\mathcal {L}_C$ for our stage 2 training could also be examined further. So far, we conducted our experiments with a decreasing consistency loss component (reducing $\alpha _e$ during training), slowly decreasing the influence of the teacher model. Other methods of modeling the teacher models influence might include increasing the consistency loss during training or keeping it constant. Examination of such effects on our method facilitates the need for further experiments. Lastly, we visually compared the GradCAM attributions for single examples. Our future work will measure and quantize the quality of attributions for both models over all images. A more rigorous investigation of this prevalent explainability method would also be desirable in this medical context.

The presented method was only evaluated on a single-center dataset. Since the method has specific requirements in regards to the quality of annotations, an external validation on CXR data is non-trivial. To the best of our knowledge, no publicly available CXR dataset meets the quality of the freehand-annotations of the dataset in this study. We conducted experiments on the CXR dataset of the 2021 SIIM-FISABIO-RSNA Machine Learning COVID-19 Challenge^44^. This dataset contains 6334 CXR images with 4 labels and bounding boxes, indicating infiltrated lung areas. We used the bounding boxes as ROI images to employ Human Knowledge Distillation equivalent to the presented study. Unfortunately, the amount of bounding boxes in the image introduced an unwanted bias to the dataset. This leads to a strong separation between classes, only from counting the bounding boxes themselves. This setup lead to a teacher model, that did not learn to use the localization of the bounding box, but rather the count of occurrence and was therefore not able to distill useful knowledge to the student model. Still, we think that external validation of our method will be important and could also be done on a different (medical) imaging domain.

While the performance of our models might not yet fulfill medical requirements for the presented study data in terms of overall performance, we argue that the improvements from applying Human Knowledge Distillation are valuable and promising. This is especially true in a medical context, where even small performance improvements are very desirable and can make a valuable difference in correct treatments. Rather than focusing on the absolute performance measures, we wanted to examine if Human Knowledge Distillation can have a positive effect on model training and performance for this study data. We think that the improvements across many models and configurations could prompt further research and adoption of our method.

## Data Availability

The data that support the findings of this study are not openly available due to relevant data protection laws for human data. A sample of the data will be made available upon reasonable academic request from the corresponding author.

## Code availability

The code associated with this paper is available on: https://github.com/dschaudt42/PneuKnowNet.
